# Consequences of Housing Conditions on Maternal Health of Forced Migrant Women

**DOI:** 10.1093/eurpub/ckac131.465

**Published:** 2022-10-25

**Authors:** M Engelhardt, M Gaudion, J Kamhiye, R Al-Munjid, T Borde

**Affiliations:** Pregnancy and Obstetric Care of Refugees, Alice Salomon Hochschule Berlin, Berlin, Germany

## Abstract

**Background:**

Housing is a major social determinant and contextual factor of health. Forced migrants are restricted in their freedom of choosing and shaping their housing conditions. This qualitative study highlights the impact of housing restrictions on reproductive and maternal health from the lived experience of forced migrant women who recently gave birth to a child.

**Methods:**

Qualitative problem-centered interviews were conducted with 33 refugee mothers (Mage = 31 years) 1-9 month postpartum. Interviewees came from 19 countries of origins, spoke 22 languages, and had lived in Germany for an average of three years. The mothers’ perspectives were complemented by 75 qualitative interviews with maternal health care professionals, (HCPs: midwives, gynecologists, social workers). Interview transcripts were analyzed via framework method regarding (1) type of housing: shared accommodation vs. private apartment, (2) region: rural vs. urban, (3) exhausting and supporting conditions as well as (4) consequences on maternal health.

**Results:**

Interviewees living in shared accommodations reported exhausting conditions hindering self-determined upbringing of their newborn, e.g., confined living spaces, racism of staff, shared kitchen and bathrooms, lack of privacy, mobility, access to medical care, hygiene. Reported consequences on maternal health ranged from physical stress to social stress and mental stress (e.g., sleeping problems, depression, fear, worrying about health of the newborn). Interviewees living in private apartments showed higher autonomy and contentment. HCPs reported missing time and staff to provide adequate support.

**Conclusions:**

Both refugee women and HCPs reported housing as main stressor during pregnancy and childbed, resulting in higher physical, social, and mental stress. Strategic social support for finding suitable private apartments for new families is needed as well as comprehensive visiting midwifery care in accommodations.

**Key messages:**

• Living conditions in shared accommodations are unacceptable from a public health and human rights perspective, especially for women during pregnancy and childbed.

• Negative effects on maternal health and self-determination of families were shown.

